# Learning analytics for lifelong career development: a framework to support sustainable formative assessment and self-reflection in programs developing career self-efficacy

**DOI:** 10.3389/frai.2023.1173099

**Published:** 2023-05-25

**Authors:** Tamishka Brass, JohnPaul Kennedy, Florence Gabriel, Bec Neill, Deborah Devis, Simon N. Leonard

**Affiliations:** ^1^University of South Australia, UniSA Educations Futures, Centre for Change and Complexity in Learning, Adelaide, SA, Australia; ^2^University of South Australia, UniSA Educations Futures, Centre for Research in Educational and Social Inclusion, Adelaide, SA, Australia

**Keywords:** machine learning (ML), learning analytics (LA), skill development, career education and development, learning feedback system, career self-efficacy (CSE), applications of artificial intelligence (AI)

## Abstract

Among myriad complex challenges facing educational institutions in this era of a rapidly evolving job marketplace is the development of career self-efficacy among students. Self-efficacy has traditionally been understood to be developed through the direct experience of competence, the vicarious experience of competence, social persuasion, and physiological cues. These four factors, and particularly the first two, are difficult to build into education and training programs in a context where changing skills make the specific meaning of graduate competence largely unknown and, notwithstanding the other contributions in this collection, largely unknowable. In response, in this paper we argue for a working metacognitive model of career self-efficacy that will prepare students with the skills needed to evaluate their skills, attitudes and values and then adapt and develop them as their career context evolves around them. The model we will present is one of evolving complex sub-systems within an emergent milieu. In identifying various contributing factors, the model provides specific cognitive and affective constructs as important targets for actionable learning analytics for career development.

## 1. Introduction

The increasing digitisation of education means we now have an abundance of data to inform educators about student learning and what is happening in the learning environment. The coupling of Machine Learning and Artificial Intelligence (ML/AI) with the capacity to gather and analyse diverse and large data sets has provided powerful ways to assess learning and impact in learning environments, and we have come to refer to this paradigm as Learning Analytics (LA) (Lang et al., 2022). The tools and approaches now available can be used by education professionals such as teachers to carry out assessment and evaluation far more efficiently. They can also be used to qualitatively operationalise different kinds of assessment and evaluation that have not been possible before, at least not at any reasonable cost.

This paper reports on early work from a project focussed on using LA to create new forms of assessment events to be embedded within a career development program for secondary school students. At this early stage of the project, we are seeking to ensure that our use of LA is primarily about learning (Gašević et al., 2014) and that the development of the computational aspects of the work to come are deliberately and deliberatively in the service of our learning goals.

The focus of the paper is a framework for using LA and ML/AI to provide effective and ongoing formative feedback to the students in our careers development program. The content under discussion here—careers education—will be of immediate interest to many readers as it is an area of teaching and learning that is currently undergoing significant curriculum reform in many parts of the world. Our primary purpose in publishing it, however, is to share our model as an example for including LA right from the initial stages of educational design. Be it for formative or summative purposes, the assessment events we create and the learning data we collect are neither neutral nor objective; they create educational realities (Perrotta and Williamson, 2018). Our purpose here is to provide a “worked example” of doing the intellectual and technical work of designing for the reality we want and choose. In the process, we are actively seeking to use the power of ML/AI to create a new educational reality in the careers development program we are designing.

### 1.1. The need for a new theory of practice in careers education

At the turn of this century, in the context of a growing policy call in many countries for the development of (lifelong) “learning societies”, Boud (2000) argued that greater attention to “sustainable” assessment was needed. Borrowing from the prevailing definition of sustainable development, he argued that meeting the assessment needs of the present should not compromise the learner's needs for the future. Moreover, he argued that the dominant summative assessment practices of the day—practices which have not, we would argue, changed all that much—were actually major barriers to quality lifelong learning.

In the two decades since, the idea of the learning society has been transformed. In the face of ever-growing automation, globalization, and collaboration, the “future of work” is emerging into an altered environment (Foundation for Young Australians, 2018) that those reading Boud's work 20 years ago could scarcely have imagined. The advent of Industry 4.0 and other digital transformations, including the pervasive use of ML/AI and automation, is causing the restructuring of many industries and the ongoing disruption of the current and future labor market (Ghobakhloo, 2020; Dvorakova, 2021). Notably in the context of our project working with secondary students, Industry 5.0 promises to disturb existing systems even further by placing research and these innovative tools and processes at the center of a shift to a sustainable, resilient, human-centric industry (Adel, 2022; European Commission, Directorate-General for Research and Innovation et al., 2022; Ivanov, 2022).

In the midst of the uncertainty for the future that these disruptions have created, our research team has been engaged in the design of career development programs for secondary school students. As this work has progressed, Boud's idea of sustainable assessment has become ever more prominent in our thinking. The idea of lifelong learning was, of course, a given. That we should support lifelong learning for young people likely to need to engage in multiple or “portfolio” careers was there in the design brief from the government-as-project-funder (National Careers Institute, 2022). As we worked to a competency-based framework however, we found that even the “light touch” assessment events we were creating had the tendency to “fragment and compartmentalize knowledge and understanding for the sake of having a manageable process which fits the time constraints” (Boud, 2000, p. 165).

This paper presents the early stages of our research and educational design intending to use LA and ML/AI to build sustainable assessment into a careers development program. The paper begins by providing further context for our educational design work, including an examination of the limitations of some existing paradigms in career education. It will then outline an alternative paradigm that can be built around the concept of career self-efficacy (CSE) (Reddan, 2015). In short, we will argue that in the absence of a clear idea of future skill needs, we - as career educators - require a different theoretical basis and framework on which to build our assessment events. We will argue that through its ability to talk to the capacity to monitor and adapt to ongoing skills-need changes (Connolly, 2020), that the concept of CSE provides the theoretical basis we are seeking. We will then articulate a framework based on this concept that can be used to translate the theory into implementable assessment events supported by LA and ML/AI. It is important to emphasize that this framework is intentionally generic. Our intention in this paper is to provide a skeleton that can evolve and adapt to many specific local contexts while enhancing students' agency in their career-based decisions through the utilization of effective LA.

## 2. Changing paradigms

As we have noted, LA and the use of ML/AI within educational assessment can be used to make existing assessment regimes more efficient, or it can be used to radically change what is being assessed. In this project we are seeking to do the latter. We are doing so because the dominant assessment form in careers education, certainly in our Australian context but elsewhere as well, continues to be based in an outdated paradigm focussed on student aptitudes and interests. In making this claim, we are not saying that aptitude and interest are irrelevant. However, as we will argue below, the dynamic nature of contemporary labor markets has seen a new paradigm develop in the relevant academic literature (Draaisma et al., 2016; Jackson and Tomlinson, 2020), in careers development policy (see, for example, National Careers Institute, 2022), and in curriculum support materials (Government of South Australia, 2022), but not yet convincingly into every-day every-school practice.

The dynamism of the contemporary labor market has been well explored (Healy et al., 2017; Connolly, 2020; Jackson and Tomlinson, 2020; Rice et al., 2022). Driven by an increasing integration of global trade and the rapid technological change, the skills-needs of most of the so-called ‘advanced' economies such as Australia have shifted toward knowledge-based and service-orientated occupations and away from manufacturing (Phelps et al., 2012; Herrendorf et al., 2014; Healy et al., 2017; Connolly, 2020; Rice et al., 2022). As this has occurred, the labor markets have become ever more ‘flexible', with a heavy reliance on casual workers and other insecure patterns of employment (Phelps et al., 2012; Herrendorf et al., 2014; Connolly, 2020). Some estimates suggest that young people finishing school in Australia today are likely to have as many as 17 different employers over a lifetime and five separate careers (Foundation for Young Australians, 2016).

In popular and policy discourse these changes are frequently presented with a mixture of wonder and alarm. For example, variations of the pseudo statistic that “N% of jobs that will exist in 20xx haven't been invented yet” are often promoted in policy documents (Government of South Australia, 2016) and by the media (e.g., Tencer, 2017; Krueger, 2021). The unfortunate consequence of this kind of reporting, however much they can only be guess work, is that it creates a perception that there is little a young person can do that will equip them for an uncertain future (McDonald, 2018; Jackson and Tomlinson, 2020). This perception is unfortunate because it simply is not true. With or without the pseudo statistics, researchers in careers development have long ago responded to what is undoubtedly an unstable and complex labor market with unpredictable skill needs by identifying an alternative paradigm for careers education (Moote and Archer, 2018). The change in paradigm we have seen has been stark.

As it evolved in the last century, the central role of careers education was to enable young people to conceptualize their interests and skills, and to use that conceptualization to provide relevant, specific and often narrowly focussed information in regard to career pathways and options (Groves et al., 2021). Often provided through one-on-one counseling, young people and their advisers sought to match personal qualities—including but not limited to, abilities, personality, and interests—against existing occupational profiles (Spokane and Cruza-Guet, 2005; Meijers and Kuijpers, 2014; Magee et al., 2022). Toward the end of last century, however, researchers began to articulate a far more expansive vision for careers education with an emphasis on the skills necessary to self-manage their own careers (see, for example, Bengtsson, 2011; Irving, 2013). This paradigm for careers education is now well entrenched in relevant policy formulation such as in the second edition of the Australian Blueprint for Career Development (National Careers Institute, 2022), which continues to direct funding in the career development space in the direction of lifelong career capabilities as did the first edition in 2010.

### 2.1. Persistent patterns of practice

The shift toward lifelong career learning has been more difficult to achieve in practice than in policy. Lifelong career learning remains a rather nebulous concept that has not yet displaced the traditional understandings that schools will prepare young people for career “pathways” associated with an ordered hierarchical progression within an organization or profession (Baruch, 2004; Jackson and Tomlinson, 2020). Put simply, in both informal discussion and more formal assessment events, we still tend to essentially ask young people “what do you want to be when you finish school?” With this underlying purpose so fixed in our practice, we focus our assessment and feedback on the destination with little space for nuance that is necessary within the complexities of the contemporary careers space. It should be of no surprise then that industry stakeholders have articulated that current career development provision is impractical, outdated and inadequately prepares students for life after school (Foundation for Young Australians, 2016; Parliament of Victoria, 2018).

In response, our project is seeking to support a different epistemology of practice (Fowler et al., 2022a,b). Rather than designing educational programs focussed on the question around the immediate issue of what comes next, we are seeking something closer to Boud's “sustainable” approach. To do so, our design is based around a different guiding assessment question: ‘how can the student improve their own CSE?'.

## 3. Our goal: feedback for career self-efficacy

The idea of CSE is not new. Developed by Hackett and Betz (1981), CSE is an adaptation of Bandura (1977) self-efficacy construct for career psychology. In Bandura's (1982) theory, self-efficacy refers to an individual's belief in their capabilities to execute courses of action, perform a given behavior, and accomplish tasks to produce specific performance attainments (Bandura, 1977, 1993, 1997; Hackett and Betz, 1981). These beliefs influence how individuals feel, think, motivate themselves and behave (Bandura, 1993). This construct can influence whether an individual chooses to perform or refrain from executing a task (Bandura, 1977, 1982), and is described as a cognitive structure shaped by cumulative learning experiences. Bandura (1977) identified four main sources of influence on self-efficacy: mastery experience; vicarious experience; social persuasion; and physiological/affective states.

While Betz and Hackett (2006) have been clear that they do not believe that CSE is a separate construct to ‘general' self-efficacy, the idea has been used to effectively draw attention to the confidence subjects have in their ability to perform the actions related to further career choices (Lent and Hackett, 1987; Anderson and Betz, 2001); and their capacity to make judgements of their abilities to perform behaviors in relation to career development and adjustment (Niles and Sowa, 1992; Anderson and Betz, 2001). Used this way, CSE provides essential information relevant to understanding the career development process (Niles and Sowa, 1992), and summarizes the possibility that low expectations of efficacy with respect to some aspect of career behavior may adversely contribute to optimal career choice (Betz and Hackett, 1986).

Deficits of self-efficacy can cause an individual to procrastinate in making career related decisions or may delay a pre-made decision from being implemented (Betz, 1992). Further, a low CSE belief, even if based on an accurate assessment of one's past accomplishments and capabilities, can hinder one's full awareness of the ability to pursue various other careers (Betz and Hackett, 1981). In contrast, the tendency to visualize success, seek positive support and outcomes for one's career ambitions, optimizes the features of high CSE. In general, the higher the CSE, the more ambitious career goals and challenges individuals will set for themselves, and the stronger their commitment will be to them (Arghode et al., 2021). As a result, low CSE beliefs should be challenged and improved, whereas high CSE beliefs should be supported and reinforced. Career development theorists have commonly acknowledged that self-efficacy beliefs are a fundamental contributor to the translating of career choice competencies into action (Taylor and Betz, 1983; Lent et al., 1994). In short, CSE beliefs can contribute to either the avoidance or motivation toward career behaviors (Betz, 2004).

The improvement of CSE has become a guiding design principle in our career development educational design project. With that in mind, we are now seeking to make effective use of LA and ML/AI to provide career educators with the pedagogical and assessment tools they need to put the development of CSE at the center of their practice. We are also seeking to do so in a way that is scalable and can support the adoption of a new kind of practice without creating a need for an additional staffing resource that is not likely to be available beyond the end of our project.

### 3.1. Learning analytics, machine learning and feedback

Our gravitation toward exploring the use of LA in our educational design work seeks to improve student CSE comes in response to recent work in the field connecting LA, decision making and self-regulated learning (see, for example, Wong et al., 2019; Blackmon and Moore, 2020). This research has taken advantage of the capacity of LA systems to provide students with meaningful feedback, otherwise expressed as “bearings” (Prinsen and de Laat, 2014; Salmon and Asgari, 2020). In this body of work, we have seen practicable solution for scaling timely and personalized feedback to support student's self-regulated learning, meeting the needs of every student in a personalized and data-oriented way (Lim et al., 2021a; Sousa et al., 2021). As Prinsen and de Laat (2014) have argued, LA can allow for students to reflect and make informed decisions as they track their achievements. In short, it is clear that student's self-assessment of their own learning can be supplemented through the use of LA (Roll and Winne, 2015), with the application of LA having been extended to decision making and interventions in the classroom (Molenaar et al., 2021).

The potential to improve feedback is important when considering how we support the development of CSE. Self-efficacy develops through mastery experiences, vicarious experiences, verbal persuasion, and the physiological information our bodies give us. Feedback, especially when targeted and personalized, can act through each of these channels. This has been most extensively explored with respect to a wider set of self-regulated learning processes (Hattie and Timperley, 2007; Lehmann et al., 2014; Lim et al., 2021a; Chung and Yuen, 2022). Of particular interest in our work is the third phase of Zimmerman's SRL cycle (Zimmerman and Moylan, 2009), which emphasizes that reflection upon and evaluation of performance and outcomes leads to more informed decisions in future learning cycles.

Previous research has demonstrated that feedback based on LA can support students' learning, regardless of prior academic standing, different learning operations and SRL competencies (Afzaal et al., 2021; Lim et al., 2021b). Afzaal et al. (2021) demonstrated, through the use of a novel data-driven approach, that LA techniques combined with explainable ML allow for automatic and intelligent feedback to be provided to students, thus facilitating the SRL process. In particular these authors showed that an ML-based algorithm, drawing on Learning Management System data to generate predictive models, allowed for data-driven feedback to be computed and actionable recommendations to be suggested.

The approach we are seeking to develop is not completely novel. Gutiérrez et al. (2020), for example, have reported on the use of analytic and predicative statistics in dashboards for learning advisers in a higher education context. Our ambition, though, is to make use of ML/AI to provide feedback directly to the students themselves and, in doing so, support higher levels of reflection and self-regulation. This approach is also part of our design brief to be scalable.

## 4. A changing framework

**Figure 1:**
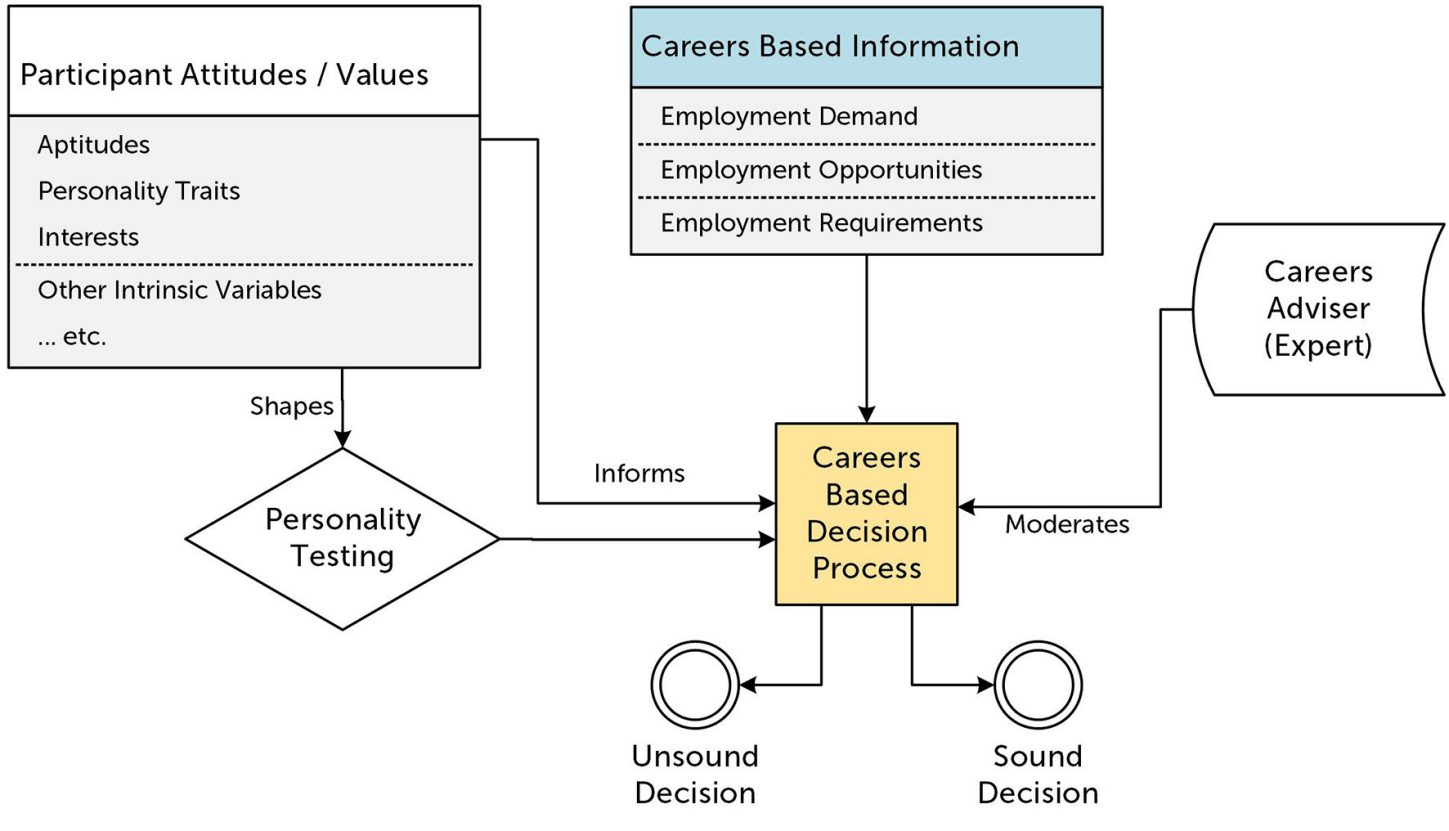
A systems framework diagram of a “traditional” careers advice conversation. Arrows indicate the flow of information in this system.

Figure 1 provides a systems overview of the framework underpinning a “traditional” approach to careers development we have described above. It essentially steps out the specification for careers advising offered by Holland (1997).

In this traditional approach the careers adviser acts as a central point of knowledge for careers-based information. From an employer or school-based perspective, this model is advantageous as there exists a central point of contact and an expert is present who can filter and process the provided data. Assisting the careers adviser in this role are the results of personality or aptitude assessments which can help in presenting a summary or subset of the available information to a specific student. However, a weakness of this approach is that it relies on research that has assumed that the dominant personality traits and aptitudes of the people currently in a given occupation are actually needed for that occupation. This may be the case but, given the cultural and social stratification of occupations where factors such as class and gender are highly determinate, it is very likely that the correlation to trait and aptitude are due to confounding, socio-economic variables.

The primary weakness we are dealing with in this paper, though, is that the process is fixed, linear and terminal. As we have argued above, this is not a suitable model for the demands of the current labor market. The model suggests that career advisers should consider a combination of trait and careers-based information available to them. Then, using some classification tools, personality testing, and/or surveys, curate a selected library of information for the student. The student should then respond to this curated information by making a decision, and the process would result in either a sound or an unsound decision being made. In many ways this process withdraws agency from the student and does not give them opportunities to autonomously explore career pathways nor develop an awareness of their CSE.

**Figure 2:**
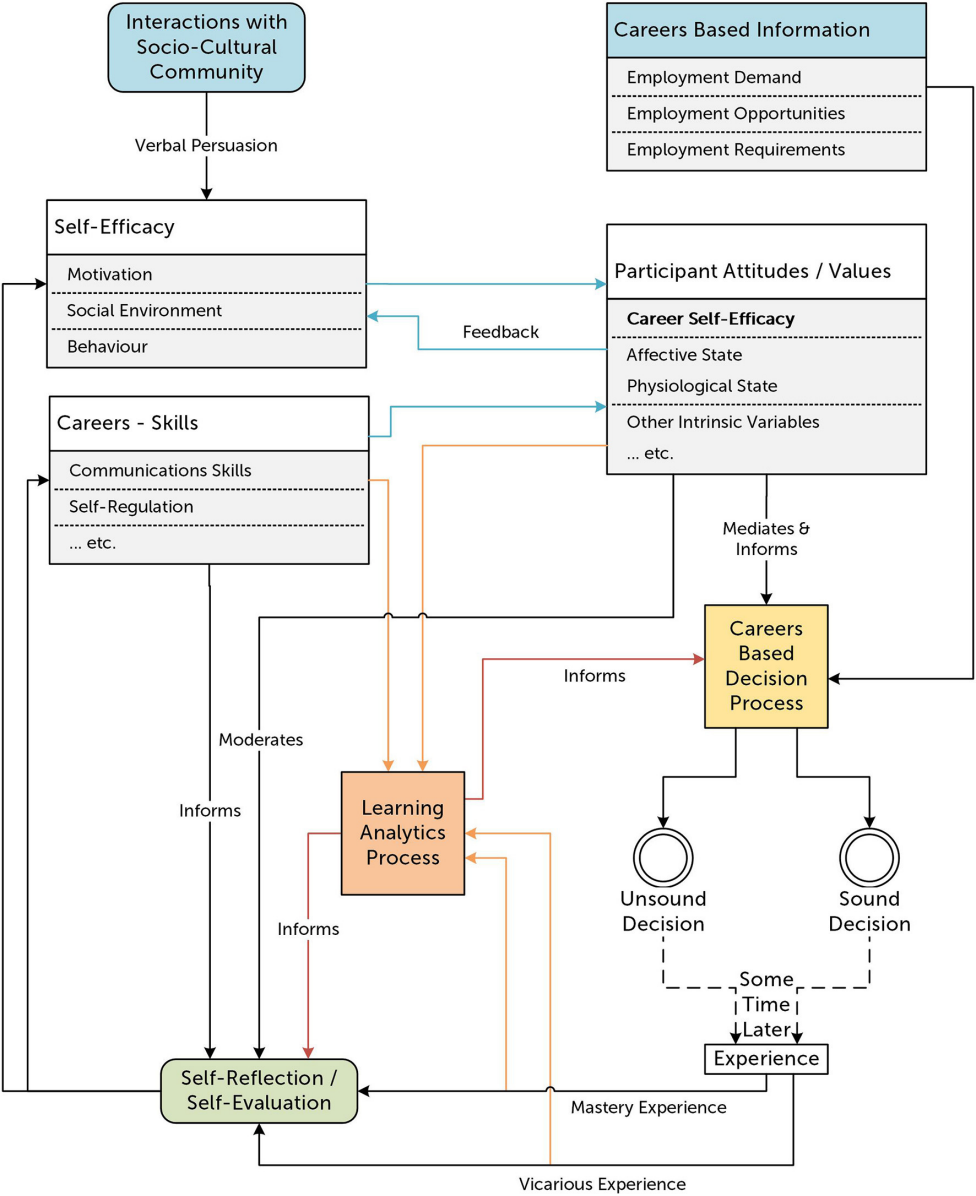
A general systems framework to explain the impact of Careers Self Efficacy on careers-based decisions. Solid black arrows indicate information flow, while dashed arrows indicate a variable time delay in this flow. Blue arrows indicate internal information flows. Orange lines indicate potential sources of data for use by learning analytics systems. Red lines indicate where data representations might add an extra “voice” to other processes.

The model set out in Figure 2 is an alternative framework informed by the ideas outlined in Groves et al. (2021). These ideas argue that the traditional careers conversation process removes autonomy from the student and instead advocate for the greater involvement of the student in the careers decision cycle. To achieve this, it is important that the student reflects both on their own mastery experiences and the vicarious experiences of others in some sort of self-reflection or self-evaluation process. In turn, this process will impact on their own underlying attitudes and values as well as their own evaluation of competencies in certain areas, including careers skills, which will mediate and inform future careers-based decisions. The framework outlined in Figure 2 incorporates these feedback loops and cycles and recognizes that careers-based decisions are in general not a “once and done” decision but rather take the form of a cumulative series of decision cycles. Careers based information in this new framework is now mediated, moderated and informed by learners' attitudes and values and the decision outcome or experience will in turn lead to further changes in these attitudes and values.

### 4.1. What is the potential role for LA in this framework?

Still a relatively young area of research, LA has tended to adopt a limited range of strategies in the provision of feedback. Excluding the approaches related to educational data mining, where the primary audience is the educational institution, LA tends toward three broad feedback mechanisms. Firstly, in the form of information visualizations either for a student or teacher audience. Secondly, in the form of feedback on teaming and other interactions from social network analysis. Finally, by the provision of “nudges” resulting from event-based triggers or content analysis (see Banihashem et al., 2022).

The role of actionable LA within the framework presented here is to support the development of CSE by providing students with data and “external” insights about their learning progress and performance. This information can help students to identify areas of strength and weakness, support them in the development of their metacognitive skills and enable them to set personal goals, and track their progress toward achieving those goals. While this information can be provided to students in any of the forms outlined above, we believe that in many cases a version of the “nudge” or prompt approach might be the most impactful. In addition, if the LA system can be used to provide personalized and targeted feedback, thus helping students to develop a greater sense of control and mastery over their learning, then their CSE can be enhanced and they will make more informed career development, and career-based decisions. This, in turn, can increase students' confidence and belief in their ability to achieve career success.

As shown by the orange arrows leading to the LA processes block in Figure 2, there are at least four data streams that can inform actionable LA systems. Specifically, there are opportunities within this model to obtain data on learners' performance, experience (both first-hand or mastery experience and vicarious experience), attitudes, and competency. The processed representations of this objective, real world-based data—in the form of dashboards, personalized prompts, or another form of appropriate feedback—can contribute another source of information, as indicated by the red arrows, to enhance a formal or informal process of self-reflection and self-evaluation. It is suggested that this reflection process will result in a shift in self-awareness of careers skills and/or self-efficacy which in turn will cause a shift in participant attitudes and values. These internal information transfers and processes are indicated by the blue arrows in Figure 2. The data representations can also be used to provide additional information at the point of decision in future careers education cycles, providing a meaningful and personalized extra “voice” in careers conversations. Using the potentially detailed insights from actionable LA in such a closed loop feedback system, it is suggested that the learner who experiences low CSE should be able to challenge their own beliefs, reflect upon their past performance and improve their attitudes and opportunities for future success.

As we move away from the specifics of this design, we can comment on the goal of the process for a moment. The process we are engaging here is one of positioning the learning first (Gašević et al., 2014). Much of the work in LA has been carried out in the context of existing educational design and has had to work with the data that is already produced by the existing designs and practices. In contrast, the need in this project to change the existing practices for secondary career development programs has provided the opportunity to include LA as a significant factor in our educational engineering. Our framework in Figure 2 incorporates a theoretical and subsequent design conjecture that specifies the place of LA as an active agent in the learning process rather than as an opportunistic “bolting-on” of LA to an existing learning system as a somewhat passive observer. That is to say that when designing new careers education programs providers need to ask the questions, “what information will the student need to gain from this experience to enhance their CSE?” and “how can the activity be built from its foundations to capture this information in a form that LA can process for the benefit of the student?”.

The cyclical nature of the framework acknowledges the complexity of an organic and evolving system and takes careers education beyond the traditional once-and-done career conversation. LA and ML/AI will be used to close the learning loops in this context. It will enable the provision of formative feedback on multiple dimensions to be delivered in real time as the student progresses through the program. This enables opportunities for our educational design team to purposefully and deliberately create spaces within the program for students to reflect on their own areas for development and, in turn, take action to build both their soft and technical skills in a fast-evolving program that reflects the fast evolution of the contemporary labor market.

### 4.2. Potential applications for this framework

While specific applications of the framework outlined in Figure 2 are beyond the scope of this paper, it may be helpful to the reader to consider two example situations where it may be utilized. As a first example, consider a careers-based program that aims to develop a student's awareness of their transferable career skills such as customer communication. The program might make use of a chatbot or other conversational agent with the intended aim being for the student to use their customer communication skills to determine the nature of the agent's inquiry and then resolve this. Naturally, the chatbot system would record the conversation which might subsequently be analyzed, using natural language processing or other techniques, to develop metrics that are relevant to the problem at hand. Using these metrics—such as communication clarity and conversation focus—the quality of the student-agent conversation, a form of mastery experience, can be measured with the data being passed into an LA Process. This LA process could be designed to provide additional feedback—in the form of a dashboard or possibly as personalized reflective prompts—to inform the student's self-reflection process. In turn this information supports the development of the student's CSE as they reflect upon their experiences, visualize their strengths, and identify the areas which may still require improvement. For example, a student who has had a good level of communication skill identified by the LA process, might receive a reflective prompt which asks them to consider possible career pathways that they believe will make effective use of this skill set. Consequently, they choose to independently research these career directions and in doing so experience enhanced CSE.

A second use case might center around the use of a reflective journal or e-Portfolio as part of a work-experience program. Under normal circumstances, a student undertaking such a program would work in isolation. However, if this framework proposed here were adopted, then this isolation could be overcome. A student undertaking such a program could be required to keep a daily reflective journal of their experiences. An ML/AI system could potentially thematically analyse the student reflections by looking for patterns and/or keywords and identify sentiment or other features of the text. These personalized analyses, a form of mastery experience, combined with the anonymised analyses of any peers undertaking a program in a similar workplace, a source of vicarious experiences, could be combined using a tailored dashboard to present the patterns in the reflections to the student. This could be in the form of generated summaries, word clouds, or other appropriate forms. Guided reflection on these data could then be used by the student to enhance their awareness of their own strengths and weaknesses and encourage the student to take positive action, thus enhancing their CSE. This authentic evidence can assist in informing self-evaluation of the work experience program and in informing future careers-based decision processes.

### 4.3. The impact of CSE?

In this framework, CSE mediates and informs the student's career-based decision process. For example, we hypothesize that a student with higher levels of CSE will: make proactive—rather than reactive—careers decisions; seek out and self-evaluate—rather than respond to provided—career-based information; be self-aware of their strengths and areas for personal improvement—rather than be oblivious of their weaknesses and developmental needs; recognize the transferability of skills between career paths—rather than considering skills as niche specialities; and consider generic career paths—rather than specific jobs. These learner characteristics describe an optimistic life-long learner and so it follows that the likelihood of making sound career decisions will be higher in students with higher CSE.

However, in order to build CSE, individuals need to be able to form a self-awareness of their career skills and effectively build their self-efficacy beliefs. This can be facilitated through the processes of self-reflection and self-evaluation, interpreting information regarding their own past performance, capabilities, and experiences—both personal and vicarious. These processes are also mediated by verbal persuasion from third parties and by affective and physiological states. Prioritizing the processes of self-reflection and self-evaluation as a focal point of investigation leads to opportunities to implement and use actionable LA to support learners in developing CSE. In our design ML/AI will provide nudges and prompt these phases of reflection.

## 5. Evaluating the framework

The framework transcends existing paradigms as it utilizes the construct of CSE as a means to inform careers-based decisions through the use of actionable LA. Current career frameworks are primarily based in the careers-based decision process box, and as the traditional conversations are a once-and-done process, there are very few opportunities to incorporate LA, and very few opportunities to learn from past decisions. However, by completing the loop, additional data streams can be captured and processed objectively by various LA processes, and then be fed back into the future decision processes of these same students. Thus, making their next decision more informed.

The cyclical framework offers numerous opportunities for multiple forms of data collection, be that from mastery and/or vicarious experiences, assessments of career skills, and surveys or instruments that measure affective attitudes. In addition, by carefully tailoring and positioning the feedback channels, these data can inform other processes within the system, potentially making the system self-moderating and damping large perturbations.

The framework acknowledges the need for learners to take charge of their own career development and promotes the increased responsibility of individuals to realize a sustainable career, aligning with the dynamic trends of current labor markets. Quite simply, the framework fosters the agency of learners to understand their own skills, capabilities, capacities, which aligns with the shift and emphasis on the importance of skills necessary to self-manage their own careers. Of course, this framework seeks to ensure that the use of LA is fundamentally about learning, and the computation aspects that follow are deliberate in the provision of specified learning goals. By using LA to help extract data from experience, and feeding that into the self-reflection and self-evaluation process, self-efficacy can be affected in a controlled way, which will affect the physiological and affective states in a controlled manner.

Furthermore, this framework encourages students to play a truly active role in the careers education process rather than the scope-limited role as seen in traditional frameworks. Within this process, there is no assessing of the student's decisions, but rather an opportunity is provided for students to self-evaluate and self-reflect on their own decisions, which in turn assists them to make informed decisions in the near future. This emphasizes how the framework contributes to students' autonomy.

By responding to the unchanged traditional career paradigm, which is still in effect to this day, the framework recognizes the long-term trajectory of the career process. With careers continually changing, progressing, and adapting to labor market demand, the ability for learners to receive targeted and personalized feedback, justifies the importance of actionable LA in the careers space, and how it can have a considerable contribution in supporting lifelong career development.

### 5.1. Potential limitations

There are some notes of caution that need to be considered with regards our proposed framework. For example, while we have identified areas in the learning system where LA will be useful, we are uncertain that we will be able to develop valid measures for the relevant variables and constructs. Furthermore, the mediating effect of intrinsic and semi-intrinsic variables such as gender and socio-economic status are likely to have additional impact on the development of these valid measures. Developments in natural language processing models in recent years combined with ever increasing efficiency in ML/AI processing give us hope that these potential technical issues are surmountable. We encourage any researchers implementing this framework to ensure that data are collected from a diverse range of participants and that an interdisciplinary team, including professional educators, is involved in discussions around the nature of any feedback from the LA processes. This will go some way toward minimizing the potential for AI bias in any application and increase the transparency and accountability of the system.

We are also not yet sure that the students will engage in the model in the way that we hope. They too are used to the orthodoxy that asks what they will be when they finish school, so they may not engage with the organic and evolving environment we are planning. Indeed, it is likely that many students will simply want an authority figure to tell them what to do. They will neither want the work that is involved in developing CSE and other complex capabilities, nor see ML/AI agents as an informed authority figure in a decision process. These issues of trust and orthodox mindset will inevitably be addressed and ultimately overcome as awareness and use of ML/AI becomes more commonplace. In the meantime, it is important that systems that make use of this framework adopt a transparent approach to the manipulation and processing of data so that both students and teachers are able to see how an LA system makes its recommendations.

Finally, we also need to test the extent to which the processes of more-or-less explicit reflection in this framework impact the import role of intuition in career decision-making (Lent and Brown, 2020). To this end, we encourage all researchers in this field to explore the impact of reflection on student values and attitudes toward careers decisions and to consider careers decision-making to be a cyclical rather than linear process.

## 6. Conclusion

The framework we have outlined in this paper is yet to be tested, but it represents an ambitious attempt to undertake educational design with LA and ML/AI in mind from the outset. As we have described, we have theoretical reason to believe that it will lead to improved practice with career education; a practice that is more aligned with recent directions in the literature and in policy.

As an initial process, however, we commend the process our project team has undertaken here for others to aspire to make use of the increasing power of ML/AI and LA to support new kinds of educational practice. The SAMR model (Blundell et al., 2022) that has been widely used when considering the role of computers in education is instructive here. It suggests that computers and other digital technologies can be used to substitute, augment, modify or redefine our educational practice. ML/AI seems the most likely technology yet to genuinely redefine practice, but doing so will require a careful and deliberate incorporation of its potential into our human-centered educational design work.

## Data availability statement

The original contributions presented in the study are included in the article/supplementary material, further inquiries can be directed to the corresponding author.

## Author contributions

TB, FG, BN, and SL led the initial conception of this paper. JK and TB developed the theoretical frameworks presented here while DD provided expertise and insight into their enaction. JK, TB, and SL led the writing of the paper and all authors contributed to the editing and refinement process. All authors contributed to the article and approved the submitted version.
